# Supplementary figures and images for: Genome-wide identification and functional analysis of circRNAs in Zea mays
Source: PLoS One. 2018 Dec 11;13(12):e0202375. doi: 10.1371/journal.pone.0202375 (PMC6289457; doi:10.1371/journal.pone.0202375)

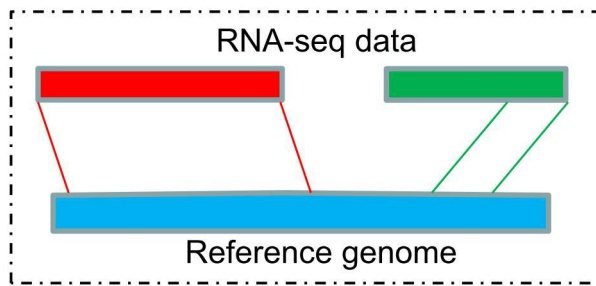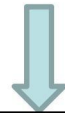

Mapping

Unmapped

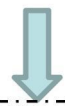

Find\_anchor

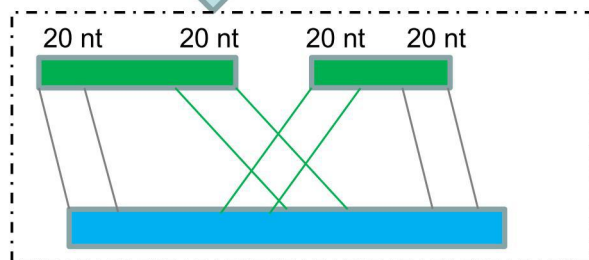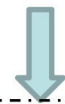

Find\_circ

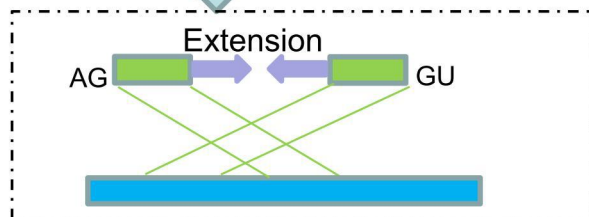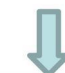

Candidates

Supplement: S1 Fig — Schematic diagram for the prediction of circRNAs in accordance with the method in a previous report. (PDF) [file pone.0202375.s001.pdf]

A

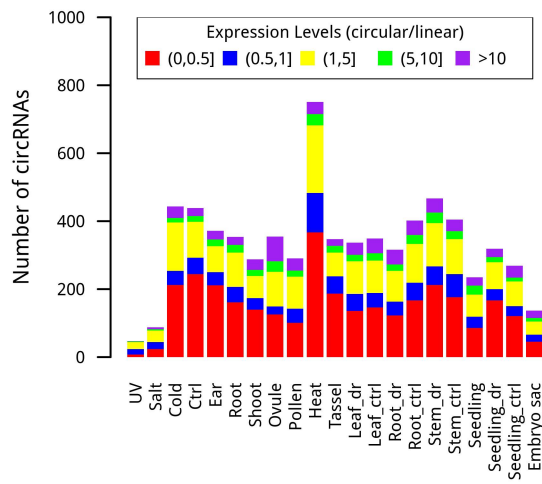

B

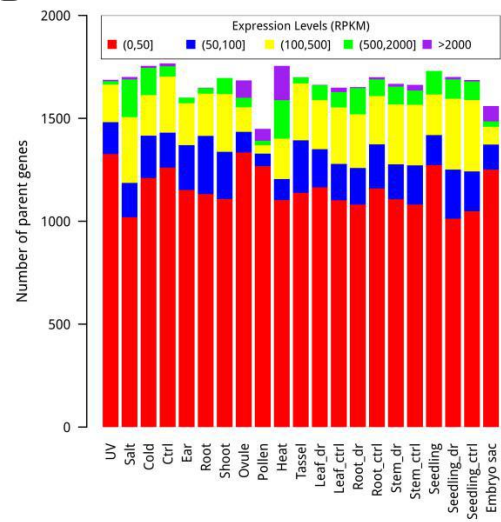

C

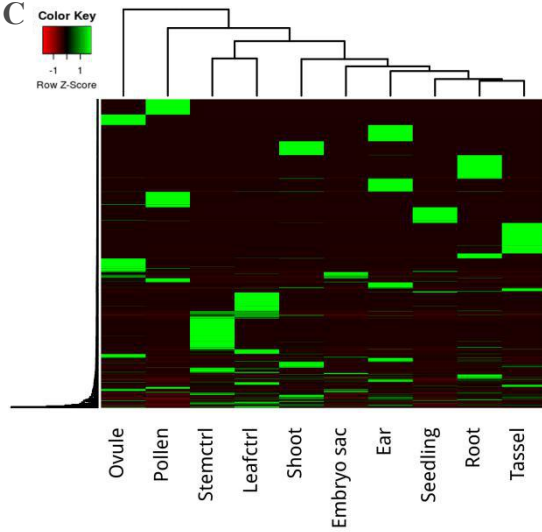

D

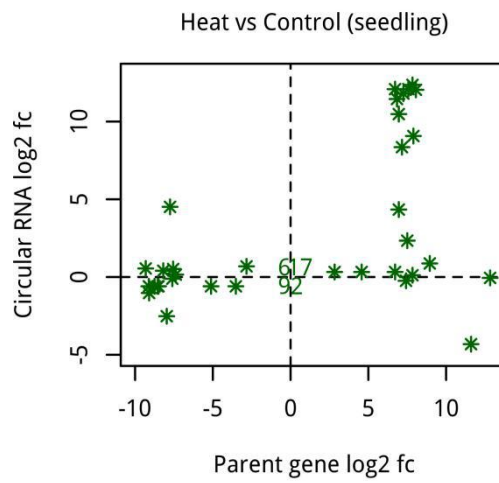

Supplement: S3 Fig — Related to Fig 3. (A) Expression level of circRNAs measured by the ratio of circular to linear in different scopes. (B) Expression level of parent genes overlapped with circRNAs in different ranges. (C) Heatmap of circRNAs with tissue-specific expression. (D) Differential expression of circRNAs and their parent genes under heat conditions in the seedlings. (PDF) [file pone.0202375.s003.pdf]

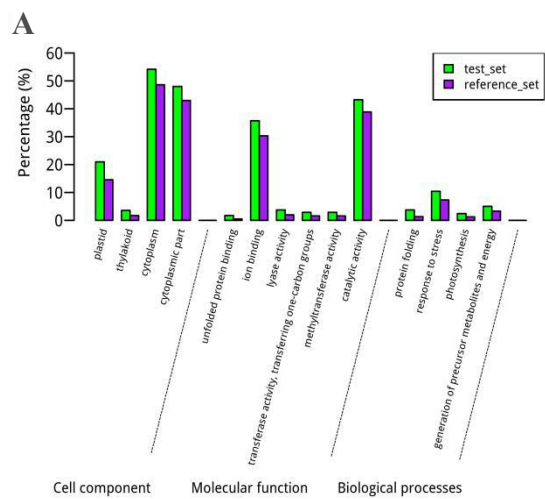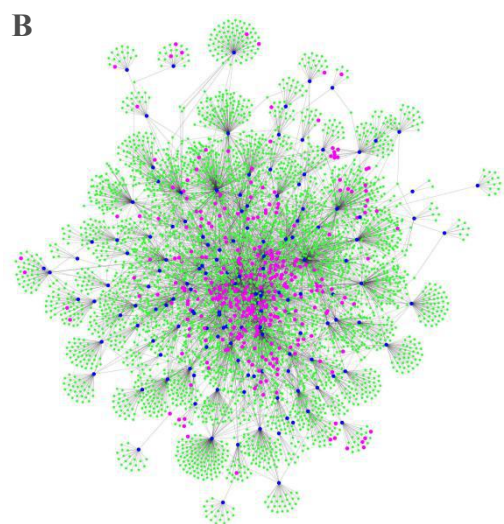

Supplement: S4 Fig — (A) Enrichment analysis for genes overlapped with circRNAs that were significant (Fisher’s test, FDR < 0.05). The x- and y-axes are the same as Fig 5. (B) Genome-wide miRNA-regulated networks. Pink nodes: circRNAs; blue nodes: miRNAs; green nodes: mRNAs. Grey edges: correlations. (PDF) [file pone.0202375.s004.pdf]
