# Supplementary material for: Genome-wide identification and functional analysis of circRNAs in Zea mays
Source: PLoS One. 2018 Dec 11;13(12):e0202375. doi: 10.1371/journal.pone.0202375 (PMC6289457; doi:10.1371/journal.pone.0202375)
Supplement: S2 Fig — (A) Example of single and multiple circularized exon and type of alternative circularization. Backspliced reads are marked. Orange rectangles: exons in transcripts; yellow rectangles: exons in another transcript derived from the same gene in the third example. Black line at the exon boundaries: backsplice site overlapped with known exon boundaries; red line: novel splice site. Absolute green arrows: backsplice using both annotated splice sites; green arrows with a blue rim: backsplice using two annotated splice sites from different transcripts; blue arrows: backsplice using no known exon boundaries. (B) Another example of sequence conservation analysis of circRNAs between A. thaliana and Z. mays. The label is the same as Fig 2C. (PDF) [file pone.0202375.s002.pdf]

A

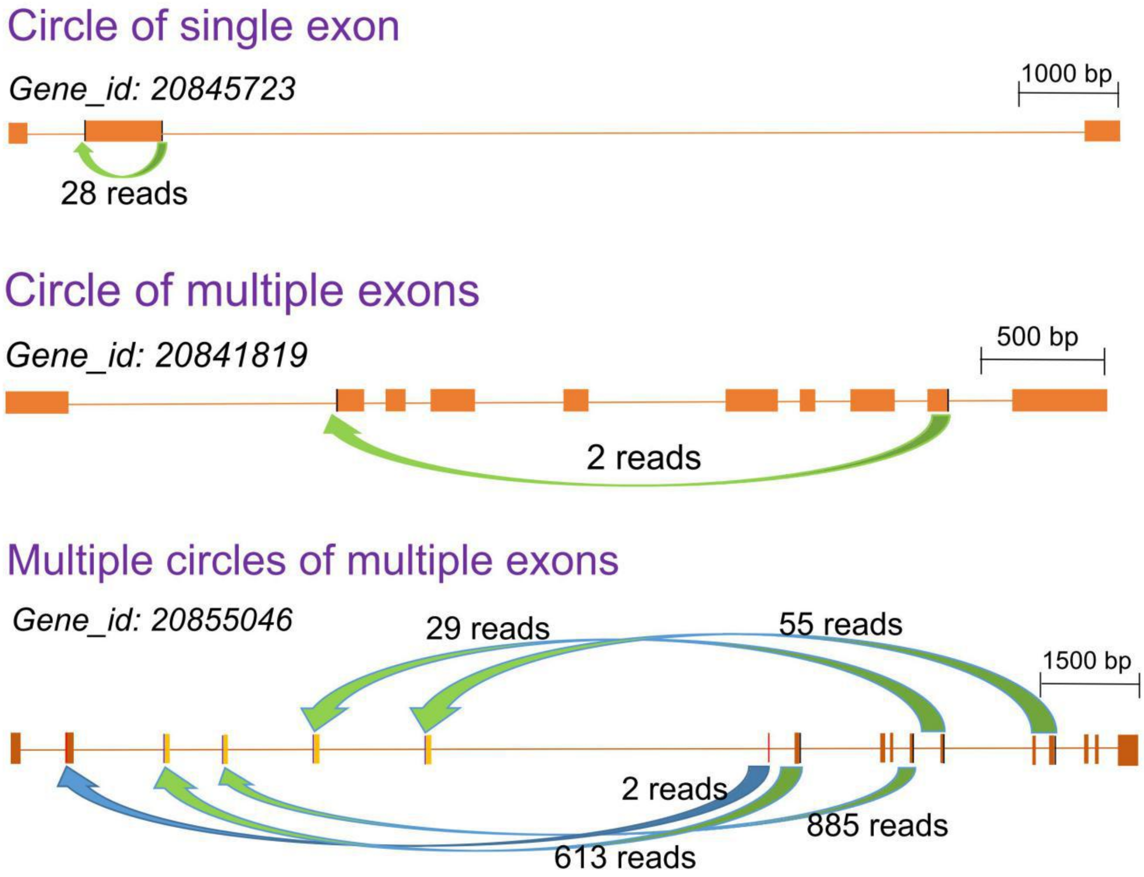

Circle of single exon
Gene_id: 20845723
1000 bp
28 reads
Circle of multiple exons
Gene_id: 20841819
500 bp
2 reads
Multiple circles of multiple exons
Gene_id: 20855046
29 reads
55 reads
1500 bp
2 reads
885 reads
613 reads

B

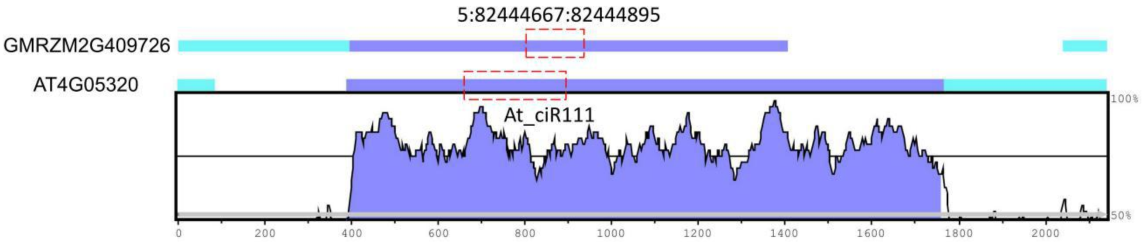

5:82444667:82444895
GMRZM2G409726
AT4G05320
At_ciR111
100%
50%
0
200
400
600
800
1000
1200
1400
1600
1800
2000
